# Do clinical outcomes in individuals with malignant gliomas differ between sexes?

**DOI:** 10.1016/j.bas.2024.104172

**Published:** 2024-12-24

**Authors:** Maria Goldberg, Laura-Sophie Frank, Ghaith Altawalbeh, Chiara Negwer, Arthur Wagner, Jens Gempt, Bernhard Meyer, Amir Kaywan Aftahy

**Affiliations:** a Department of Neurosurgery, School of Medicine, Klinikum rechts der Isar, Technical University Munich, Munich, Germany; b School of Medicine, Technical University Munich, Munich, Germany; c Department of Neurosurgery, University Medical Center Hamburg-Eppendorf, Hamburg, Germany

**Keywords:** High-grade glioma, Gliosarcoma, Diffuse astrocytoma, Anaplastic oligodendroglioma, Sex differences

## Abstract

**Introduction:**

Sex-related differences in the epidemiology of malignant gliomas are acknowledged; however, information regarding their clinical characteristics and outcomes after surgery is limited.

**Research question:**

To identify sex-specific differences of all patients with high-grade glioma at our institution and assessed clinical outcomes and prognostic factors.

**Material and methods:**

This single-center study included those who underwent surgery for malignant gliomas between 2010 and 2020. Categorical, normally distributed, and skewed continuous variables were compared between men and women using the chi-square test, independent samples *t*-test, and Mann–Whitney *U* test, respectively. Survival was calculated using the log-rank and Kaplan–Meier methods.

**Results:**

In total, 621 patients with WHO grade IV gliomas were identified (370 (59.58%) male). Men were significantly younger, underwent surgery faster after imaging diagnosis, and had a slightly higher surgical complications incidence than women. Women reported a worse preoperative performance status. Multivariate analysis showed that sex did not affect survival, surgical complications, nicotine or alcohol abuse, or preoperative tumor volume. Age, Karnofsky performance status, neurosurgical resection, and adjuvant radiotherapy with temozolomide showed a survival advantage.

**Discussion and conclusions:**

Men are diagnosed with malignant glioma at a younger age than women; however, no advantage in clinical outcomes was observed. No sex-related differences were observed.

## Highlights

• Study shows no sex-based advantage in survival rates for malignant glioma patients, despite men being diagnosed younger and fitter.
• Key survival determinants include age, performance status, and treatment approach, not patient sex.
• Findings highlight equality in clinical outcomes between males and females post-surgery.

## 1 Introduction

Gliomas are the most common primary brain tumors, with highly malignant glioblastomas accounting for almost half of the diagnoses (Ostrom et al., 2019). The median survival of patients with gliomas is extremely poor; approximately 3 months when not treated (Thakkar et al., 2014). However, the overall survival can be prolonged for up to 16 months for patients who receive maximal multimodal treatment and initially have a better functional status (Gilbert et al., 2013, 2014; Stupp et al., 2005; Chinot et al., 2014). Thus, the identification of favorable prognostic factors remains an important aspect of cancer research.

Based on epidemiological data, women have a lower incidence of cancer and better therapeutic response (Micheli et al., 2009; Siegel et al., 2017). A similar trend has been observed in neurooncology wherein the ratio of males to females diagnosed is estimated to be approximately 1.5:1 (Louis et al., 2021). In addition to the difference in incidence, patient outcomes vary, with women showing better overall survival (Trifiletti et al., 2017; Frandsen et al., 2018). The clinical presentation may also differ, with a higher rate of epileptic seizures reported in males (Ranjbar et al., 2019). However, a retrospective study reported a larger tumor size and necrotic area in females (Bilello et al., 2016). Several studies have also suggested differences in anatomical location between men and women (Bilello et al., 2016; Li et al., 2018). The protective effect of estrogen has also been suggested as a favorable factor owing to the increased risk of high-grade glioma associated with a later age of menarche (Hatch et al., 2005; Felini et al., 2009). In contrast, some studies have suggested an association between male hormone exposure and tumor development based on in vivo and in vitro experiments (Hopewell, 1970; Yu et al., 2015). Additionally, differences in neuroimmunological tumor responses are suggested to contribute to variations in treatment response and tumor growth (Carrano et al., 2021) with standard therapy being more effective in women, which is associated with the expression of integrin signaling pathways, based on transcriptomic analysis (Yang et al., 2019).

Despite evidence regarding varied incidences and better survival rates, the prognostic factors for patients with gliomas remain unclear. Moreover, evaluation of the differences in clinical factors and treatment outcomes has the potential to improve our understanding of the disease course and treatment response. Consequently, here we report a sex-specific analysis of all patients who underwent surgery for high-grade glioma at our institution and assessed clinical outcomes and prognostic factors.

## 2 Methods

All patients with high-grade gliomas Grade IV diagnosed between 2010 and 2020 at our institution were retrospectively analyzed. Histological diagnoses included glioblastoma, gliosarcoma, diffuse astrocytoma, anaplastic oligodendroglioma, diffuse midline glioma, and anaplastic astrocytoma. All histopathological diagnoses were revised based on the 2016 WHO classification. Clinical information was retrieved from the hospital server and analyzed for the entire cohort, subdivided by sex. The following characteristics were analyzed: age, risk factors, preoperative and postoperative tumor volumes, anatomical localization, extent of resection, Karnofsky performance status before and after surgery, overall survival, recurrence-free survival, and adjuvant treatment.

T_1_-weighted MRI images were used for volumetric analysis to identify contrast-enhanced regions using Origin®Software from Brainlab (Ver. 3.1; Brainlab AG, Munich, Germany).

This study was conducted in accordance with the ethical principles of the Declaration of Helsinki. The study protocol was approved by the local ethics committee of the Technical University of Munich (approval number 5625-12). The requirement for written informed consent was waived by the Ethics Committee due to the retrospective nature of the study.

Statistical analyses were performed using SPSS (version 22.0 (SPSS Inc., Chicago, IL, USA) and GraphPad Prism (ver. 8.3.1332). Continuous and categorical variables were expressed as mean ± standard deviation (SD) and frequency (percentage), respectively. Categorical, normally distributed, and skewed continuous variables were compared between men and women using the chi-square test, independent samples *t*-test, and Mann–Whitney *U* test, respectively. Survival was calculated using the log-rank and Kaplan–Meier methods. Statistical significance was set at P < 0.05.

## 3 Results

A total of 621 patients with WHO Grade IV gliomas were identified in this study. The histological diagnoses and anatomical localization are presented in Table 1.

**Table 1:** Histological diagnoses and anatomical localization of patient samples.

Histology	Male N (%)	Female N (%)
glioblastoma	334 (90.27%)	223 (88.84%)
gliosarcoma	6 (1.62%)	9 (3.59%)
diffuse astrocytoma	9 (2.43%)	2 (0.80%)
anaplastic oligodendroglioma	4 (1.08%)	6 (2.39%)
diffuse midline glioma (H3.3 K27M-mutant)	3 (0.81%)	1 (0.40%)
malignant glioma undefined	12 (3.24%)	9 (3.59%)
anaplastic astrocytoma	2 (0.54%)	1 (0.40%)
**Tumor localization**		
frontal	83 (22.43%)	54 (21.51%)
temporal	93 (25.14%)	52 (20.72%)
occipital	32 (8.65%)	17 (6.77%)
central	30 (8.11%)	22 (8.76%)
corpus callosum	20 (5.41%)	18 (7.17%)
parietal	36 (9.73%)	31 (12.35%)
multilocular	24 (6.49%)	22 (8.77%)
insula	30 (8.11%)	17 (6.77%)
Brainstem & basal ganglia	6 (1.62%)	4 (1.59%)
thalamus	7 (1.89%)	8 (3.19%)
limbic	3 (0.81%)	5 (1.99%)
cerebellar	3 (0.81%)	1 (0.40%)
ventricular	3 (0.81%)	0 (0.00%)

Molecular data on available markers, including IDH1 mutation status, MGMT-promoter methylation status, Ki67, EGFR, and p53 expression from the period 2010–2020 is summarized in the following Table 2.

**Table 2:** Distribution of molecular markers in male and female patients.

	Male	Female	P
IDH1 R132H positive	285 (No, 77.03%), 14 (Yes, 3.78%), 71 (NA, 19.19%)	187 (No, 74.50%), 9 (Yes, 3.59%), 55 (NA, 21.91%)	0,708972595
MGMT-promotor	188 (Unmethylated, 50.81%), 96 (Methylated, 25.95%), 86 (NA, 23.24%)	85 (Unmethylated, 33.86%), 93 (Methylated, 37.05%), 73 (NA, 29.08%)	0,000134882
Ki67 (%)	35.88 ± 19.74	33.04 ± 19.08	0,114743465
EGFR	42 (Negative, 11.35%), 159 (Positive, 42.97%), 169 (NA, 45.68%)	22 (Negative, 8.76%), 95 (Positive, 37.85%), 134 (NA, 53.39%)	0,153112503
p53 (%)	55.19 ± 36.49	46.48 ± 36.86	0,110249455

**Fig. 1:**
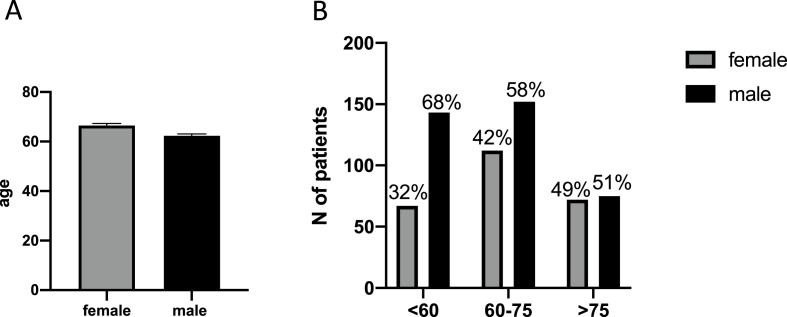
Age distribution at the time of diagnosis.

Among the patients 621 patients, 370 (59.58%) were male and 251 (40.42%) were female with a ratio of 1.4:1. Median age at the time of diagnosis was 69 for women and 64 for men patients (Fig. 1A, p = 0.000248). There were significantly more men in the age category <60 years old and in 60–75 years old, with only 32% and 42% of women being diagnosed with a high-grade glioma in these age groups, respectively (Fig. 1B, p = 0.0036).

**Fig. 2:**
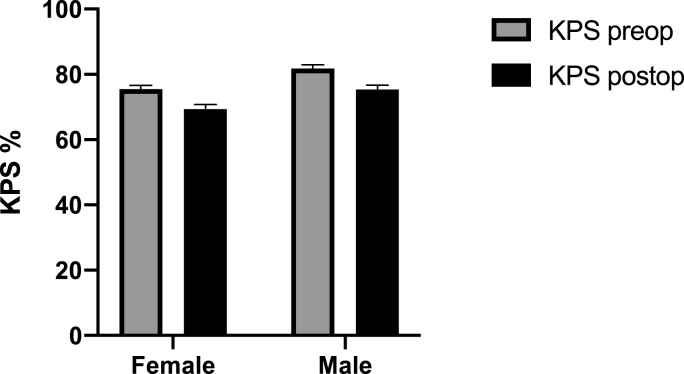
Preoperative and postoperative performance status.

A significant difference was observed in Karnofsky performance scale scores between the sexes with preoperative mean for men vs women being 80,65 ± 16,69 vs 75,46 ± 18,55 (Fig. 2, p = 0.001).

**Fig. 3:**
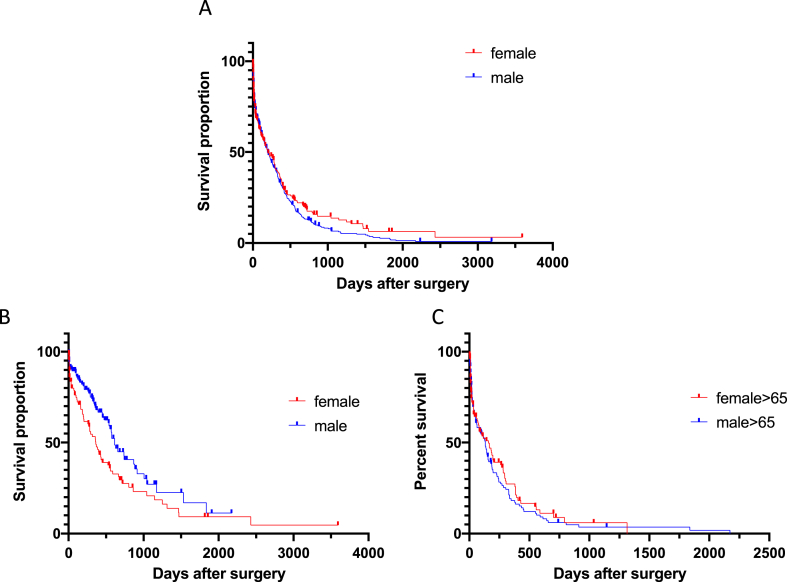
Overall survival after surgery.

No difference was observed in preoperative tumor volume (33,7 ± 37,72 vs 36,64 ± 33,89, p = 0.328103). Women underwent surgical treatment after diagnosis significantly later than men with a delay of approximately 10 days (34,64 ± 158,63 vs 24 ± 53,94, p = 0.006371). Overall survival of men and women post-surgery was 289.5 ± 397.72 and 273.26 ± 430.98 respectively (Fig. 3A, p = 0.2041).

Total resection was achieved in 135 (36.49%) men and 88 (35.06%) women (P = 0.1964). Despite no significant difference in OS, men tended to benefit more after complete resection than women (Fig. 3B, p = 0.0093). However, when only older patients were compared, no significant difference was observed (Fig. 3C, p = 0.4898).

**Fig. 4:**
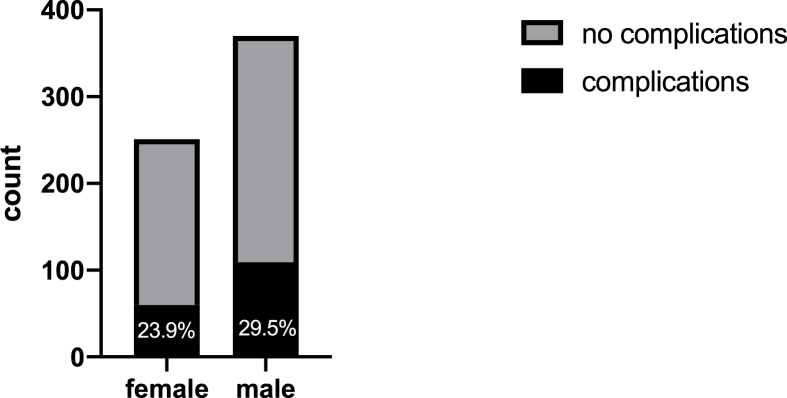
Percentage of surgical complications.

Additionally, men tended to have more surgical complications than women. Postoperative complications were observed in 109 men (29.5 %) and 60 women (23.9 %) (Table 3 and Fig. 4; p = 0.1296).

**Table 3:** Postoperative complications.

	Male N (%)	Female N (%)
**Complications, n (%)**	109 (29.46%)	60 (23.90%)
bleeding	30 (8.11%)	28 (11.16%)
abscess	9 (2.43%)	1 (0.40%)
Hydrocephalus requiring a shunt	22 (5.95%)	14 (5.58%)
CSF leakage	16 (4.32%)	7 (2.79%)
infection	28 (7.57%)	8 (3.19%)
EVD	27 (7.30%)	23 (9.16%)
seizure	13 (3.51%)	7 (2.79%)
sedation	7 (1.89%)	2 (0.80%)
dislocation of bone flap	30 (8.11%)	24 (9.56%)
hygroma	0	2 (0.80%)
SMA symptoms	7 (1.89%)	0
edema	8 (2.16%)	0
death	11 (2.97%)	11 (4.38%)

**Fig. 5:**
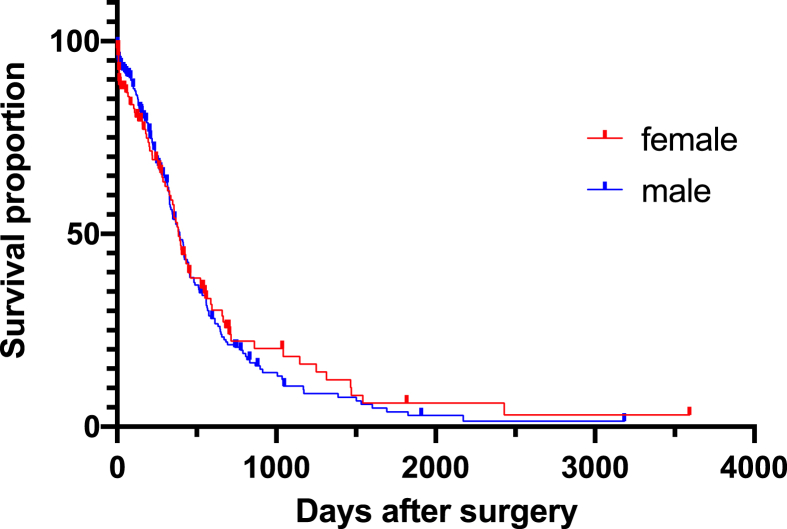
Survival after adjuvant radio- and chemotherapy.

In total, 237 (64.05%) men and 126 (50.20%) women received standard treatments including adjuvant radiation and chemotherapy with temozolomide. However, no difference in survival was detected between the two groups (Fig. 5, p = 0.941).

**Fig. 6:**
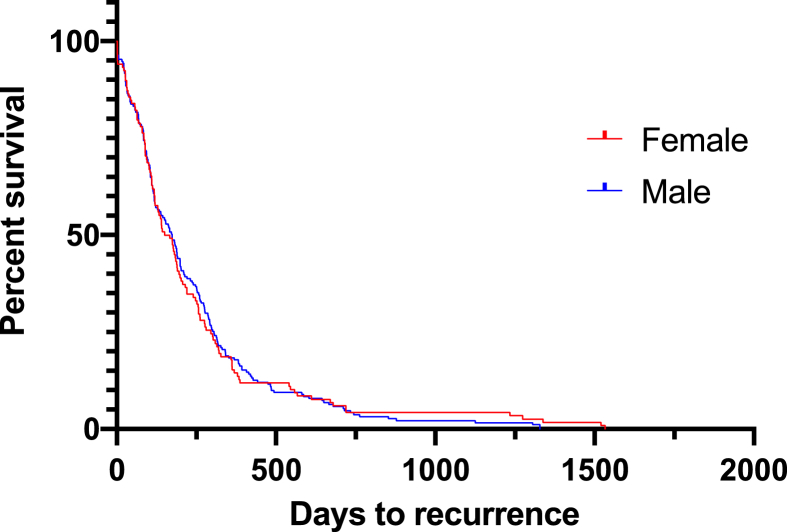
Time to recurrence.

Recurrence was observed in 191 men (51.62%) and 118 women (47.01%) with no difference in the time to recurrence (Fig. 6, p = 0.9295).

Multivariate analysis included the following factors: age, sex, preoperative KPS, preoperative tumor volume, nicotine and alcohol abuse, tumor recurrence, surgical complications, and adjuvant radiotherapy and chemotherapy with temozolomide (Table 4). Sex, surgical complications, nicotine or alcohol abuse, and preoperative tumor volume did not affect patient survival. In contrast, known prognostic factors such as better preoperative functional status, total resection, younger age, tumor recurrence, and adjuvant therapy showed a survival advantage.

**Table 4:** Multivariate analysis of risk factors associated with survival.

Risk factor	Hazard ratio	Lower 95% CI	Upper 95% CI	P value
Sex (female)	0,85	0,69	1,04	,117
**Age**	**1,04**	**1,03**	**1,05**	**< .001**
**Surgery (total resection)**	**2,01**	**1,5**	**2,7**	**< .001**
Tumor volume (mm^3)^	1	1	1	,368
Nicotine abuse (yes)	1,58	0,84	2,97	,158
Alcohol abuse	1,13	0,58	2,23	,715
**Preoperative KPS (70**–**100)**	**0,99**	**0,98**	**0,99**	**< .001**
Surgical complications (yes)	0,91	0,71	1,15	,422
**Recurrence (yes)**	**2,56**	**2,06**	**3,18**	**< .001**
**Adjuvant radiation and temozolomide treatment (no)**	**1,62**	**1,29**	**2,04**	**< .001**

## 4 Discussion

Sex differences in high-grade gliomas have been investigated to improve prognostic evaluation and treatment management (Matteoni et al., 2020) and occur more often in men than in women (Kim et al., 2018). The p53 functional status differs between male and female; hence, one explanation is that the loss of p53 function increases the risk of glioma development (Sun et al., 2014; Haupt et al., 2019). Another theory highlights the potentially protective role of estrogens (Kabat et al., 2010). Consistent with the above results, we observed a higher incidence of high-grade gliomas in males with the ratio of 1.4:1. It has also been reported that women are diagnosed and undergo surgery at an older age than men (Tavelin and Malmström, 2022), this was similar to the findings observed in our study. Moreover, the younger cohort had more men.

The MGMT promoter methylation status has been studied extensively for its impact on glioma prognosis and treatment response. A key finding is that MGMT promoter hypermethylation is linked to a more favorable prognosis in glioblastoma, particularly when patients are treated with alkylating agents like temozolomide (Weller et al., 2011). MGMT methylation often predicts better overall survival and progression-free survival (Sarathy et al., 2019). Interestingly, there are notable gender differences in the prevalence of MGMT methylation. Some studies have reported higher methylation levels in female glioblastoma patients compared to males, suggesting potential gender-related biological differences that might influence treatment outcomes (Zawlik et al., 2009). This observed difference in methylation status between genders could correlate with the reported higher percentage of MGMT promoter hypermethylation in females, which in turn may result in better treatment responses or outcomes. Furthermore, while MGMT methylation status generally correlates with improved survival outcomes, studies have noted its lack of prognostic significance in certain demographics, indicating that its predictive power may be context-dependent (Jovanović et al., 2019). This stresses the importance of considering demographic factors like age and gender in research and clinical practice. Additionally, MGMT methylation is also associated with other molecular markers, such as IDH mutations, that could provide a more detailed understanding of glioma biology and its implications for different patient groups (Ohka et al., 2011). In summary, the differences in MGMT promoter methylation between genders could potentially explain some variance in the clinical outcomes seen in glioma patients. Understanding these differences might help tailor more effective treatment strategies. For further insights into specific mechanisms and broader implications, one might need to explore the interaction between MGMT methylation and other genetic and epigenetic factors (Zheng et al., 2009). In our study, we also noticed that a higher proportion of men had an unmethylated MGMT promoter compared to women, which might contribute to the observed gender differences in treatment response and clinical outcomes.

Differences in the anatomical predispositions have been reported by Li et al. More lesions were observed in frontal lobe rather than in temporal in men when compared to women (Li et al., 2018). However, we did not observe any differences in the anatomical localization. In one study, volumetric analysis revealed a larger lesion size and larger necrotic area in women (Bilello et al., 2016), but based on our analysis, no differences in tumor volume at the time of diagnosis could be identified.

A higher KPS is a known favorable prognostic factor in neurooncological research (Asklund et al., 2015). Tavelin et al. reported significantly lower preoperative functional status in female patients (Tavelin and Malmström, 2022), similar to what was observed in this study. Studies have shown that men have a higher risk of death and worse overall survival compared to women (Gittleman et al., 2019; Stabellini et al., 2021). However, our data do not support these findings, showing similar survival rates between sexes.

Stabellini et al. reported a higher percentage of men received multimodal treatment compared to women. Moreover, men tend to undergo surgical resection later then women (Stabellini et al., 2021). Our observations showed a similar trend, with more men undergoing multimodal surgery and adjuvant therapy. In our study, however, we observed that women presented with significantly longer waiting times compared to men, which may be influenced by various socio-cultural factors such as health-seeking behaviors, availability of support networks, and differences in socio-economic status. The understanding and addressing of socio-cultural factors are crucial in elucidating gender differences in healthcare access and outcomes. Various studies have underscored the role of socio-cultural dynamics and their impact on healthcare for both men and women. For instance, healthcare utilization patterns often differ significantly between genders due to socio-demographic and economic factors. Women may delay or avoid seeking healthcare due to competing family roles or economic constraints, such as lack of insurance coverage or difficult access to clinics (Lieberman et al., 1997). Additionally, a study highlighting gender differences in healthcare utilization among Medicare-aged Americans suggests that women are less likely to use certain health services like hospital care and outpatient surgery but are more inclined to use home care services, reflecting socio-cultural influences on health-seeking behaviors (Song et al., 2006). Gender differences are also exacerbated by socio-cultural expectations and roles. For example, a study on healthcare utilization in the United States explores how employment and having children influence healthcare access, noting that such socio-cultural factors significantly affect physician visits and healthcare outcomes (Xu and Borders, 2003). Another study reveals significant gender and ethnic/racial disparities in healthcare access among older adults, further supporting the idea that cultural factors beyond economic access contribute to these differences (Dunlop et al., 2002). Therefore, it is vital to consider these socio-cultural factors in the healthcare discussion to develop more equitable and culturally sensitive healthcare policies. Addressing these disparities calls for multifaceted interventions tailored to the socio-cultural contexts of different populations (Bacigalupe et al., 2022).

The extent of resection is a significant prognostic factor (Kotrotsou et al., 2018) with women showing slightly better survival rates when complete resection is achieved (Tavelin and Malmström, 2022). Nevertheless, surgical resection is associated with a significant risk of postoperative complications and reduced functional status (Sawaya et al., 1998). Similar total resection rates were observed in our study; however, men had higher postoperative complication rates. Moreover, men showed better survival after complete resection than women. The younger age of male patients in our cohort was suggested as the main factor for better outcomes because this difference was not observed when comparing older patients alone.

Several authors have reported better treatment responses in women after multimodal treatment with adjuvant radiation and temozolomide (Johansen et al., 2020; Gupta et al., 2013); no difference in overall survival was observed between the groups after the standard treatment in our study. Time of recurrence is an independent prognostic factor associated with extremely poor survival (Audureau et al., 2018); no differences in relapse time or recurrence rate were observed in the current study.

Moreover, multivariate analysis showed that sex did not play a prognostic role in patients with high-grade gliomas. As previously shown, factors such as age, total resection, multimodal adjuvant treatment, tumor relapse, and functional status can be used as prognostic factors.

Integrating insights from extensive research highlights the necessity for personalized treatment plans that consider these diverse factors, refining approaches to glioma management across different patient demographics.

### 4.1 Limitations

Due to the retrospective design of the study, missing information on confounders could have led to bias. Another limitation was the lack of homogeneity, particularly in terms of histopathological diagnoses. Although all tumors were reclassified based on the WHO 2016 classification, data on molecular characteristics were missing, which could add value to understanding the differences in the disease course. Therefore, a prospective study accounting for the precise molecular features of the tumor would allow for a better evaluation of sex-related differences and more personalized approaches.

## 5 Conclusions

Higher proportion of men were diagnosed with high-grade gliomas, were younger at the time of diagnosis, and had more surgical complications than female patients, whereas women had surgical treatment later and had a lower preoperative functional status. However, these facts do not provide an advantage for survival.

## Data sharing statement

The datasets used and/or analyzed during the current study areavailable from the corresponding author upon reasonable request.

During the preparation of this manuscript, the authors utilized the services of ChatGPT to assist with language editing. Following this, the authors thoroughly reviewed and edited the content as necessary, and assume full responsibility for the publication's content.

## Funding

The research received no specific grant from any funding agency.

## Declaration of competing interest

The authors declare that they have no known competing financial interests or personal relationships that could have appeared to influence the work reported in this paper.
